# Pien-tze-huang promotes wound healing in streptozotocin-induced diabetes models associated with improving oxidative stress *via* the Nrf2/ARE pathway

**DOI:** 10.3389/fphar.2023.1062664

**Published:** 2023-01-12

**Authors:** Ying Liu, Jiake Mo, Fang Liang, Siwei Jiang, Jing Xiong, Xubiao Meng, Zhaohui Mo

**Affiliations:** ^1^ Department of Endocrinology, Third Xiangya Hospital of Central South University and Diabetic Foot Research Center of Central South University, Changsha, Hunan, China; ^2^ Department of Endocrinology, Haikou people’s Hospital & Haikou Affiliated Hospital of Central South University Xiangya School of Medicine, Haikou, Hainan, China

**Keywords:** pien-tze-huang, diabetic wound, Nrf2, oxidative stress, inflammatory

## Abstract

Diabetic foot ulcers are a serious complication of diabetes, with high mortality and a lack of effective clinical treatment, which leads to a considerable financial burden. Pien-Tze-Huang (PZH) is a Chinese traditional medicine with a long history that has been found to be an effective and convenient treatment for inflammatory diseases such as skin abscesses and ulcers. In this study, we assessed the effects of PZH on diabetic wounds and the underlying mechanisms. The wounds were established on the backs of streptozotocin-induced type 1 diabetic rats and type 2 diabetic mouse models. We found that PZH treatment used locally or by gavage significantly promoted wound healing, accelerated re-epithelialization and vasculature in the wound tissue, upregulated the expression of the growth factors VEGF-A, PDGF, and EGF, and activated the Nrf2/ARE pathway in the wound tissue. *In vitro* assays showed that PZH improved the proliferation, migration and angiogenic function of human umbilical vein endothelial cells (HUVECs) cultured in palmitic acid, reduced the expression of the apoptotic proteins p53, Bax, and cleaved-caspase3, and activated Nrf2/ARE signaling; however, these protective effects were abrogated after Nrf2 was knocked down by specific siRNA. In addition, the levels of the serum inflammatory cytokines IL-1β, TNF-α, and IL-6 were reduced after PZH gavage treatment. In conclusion, the positive role of PZH in diabetic wound healing might be related to the activation of the Nrf2/ARE pathway to regulate the level of oxidative stress *in vivo* and increase the expression of growth factors to improve angiogenesis.

## 1 Introduction

Diabetic foot ulcers are one of the most common and serious complications of diabetes (Lavery et al., 2006). The annual incidence of diabetic foot ulcers is approximately 2%, with a lifetime prevalence ranging from 19% to 34%. Diabetic foot ulcers usually have poor healing outcomes. Approximately 20% of moderate and severe diabetic foot ulcers may suffer from infection and angiogenesis disorders, resulting in poor wound healing and eventual amputation, which is the leading cause of non-wound amputation in most countries and can be fatal in severe cases (Hao et al., 2014; Armstrong et al., 2017). The recurrence rate can be as high as 40%, and the cost of treatment is expensive (Boulton et al., 2005). The treatment of diabetic foot is still a global challenge, and new treatment strategies are urgently in demand.

In recent years, traditional Chinese medicine has attracted attention in this field because of its various active ingredients, which can promote diabetes wound healing through multiple signaling pathways simultaneously to take excellent and safe curative effects (Zhou et al., 2022). The natural healing process of wounds involves a complex series of cellular and molecular processes, any of which can be disrupted, resulting in impaired wound closure (Falanga, 2005; Reiber and Raugi, 2005; Janis and Harrison, 2016). Oxidative stress plays a crucial role in the wound healing of diabetes. The imbalance of the free radicals production and antioxidants in the body will lead to excessive production of reactive oxygen species (ROS), which will lead to cell and tissue damage and delayed wound healing (Avila et al., 2013; Eming et al., 2014). Therefore, reducing ROS levels through the antioxidant system can reduce oxidative stress induced damage to improve healing.

Pien-Tze-Huang (PZH), a well-known Chinese traditional medicine with a long history in China, its formula is a state protected secret which mainly based on Panax notoginseng (*Araliaceae*; Notoginseng radix et rhizoma; Sanqi in Chinese; 85%), Snake bile (*Colubridae*; Bile juice of snake gallbladders; Shedan in Chinese; 7%), Bezoar bovis (*Bovidae*; Dried cattle gallbladder stones; Niuhuang in Chinese; 5%), and Moschus (*Cervidae*; Excretion of Moschus berezovskii Flerov, Moschus sifanicus Przewalski, or Moschus moschiferus Linnaeus; Shexiang in Chinese; 3%) (Zhang et al., 2022), which relieve heat, detoxify, activate blood circulation and remove blood stasis, reducing swelling and pain in the theory of traditional Chinese medicine (Qiu et al., 2018). PZH is usually used for treating inflammatory diseases and tumors (Qi et al., 2016; Chen et al., 2017; Li L. et al., 2018; Deng et al., 2020). Although PZH has been used in treating folliculitis and furuncle for many years, the research about PZH treating diabetic foot ulcers is limited. Recently, a study revealed the possibility of PZH in treating diabetic wounds associated with its antioxidant function (Yan et al., 2020), but the underlying mechanism remains unclear.

The nuclear factor erythroid-associated factor 2 (Nrf2) is one of the major contributors to the protection and restoration of cellular homeostasis, it is activated by cellular stressors, including ROS, xenobiotics, electrophilic toxicants, and the dissociation from Kelch-like ECH- associated protein 1 (Keap1), triggering the transcription of more than 200 downstream endogenous protective genes, including those encoding antioxidants, detoxifying enzymes, antiapoptotic proteins, proteasomal genes and anti-inflammatory costimulatory genes (Niture et al., 2014). The data showed that Nrf2 activity was damaged in diabetic patients, the wound healing was delayed in Nrf2^−/−^ diabetic mice, and accompanied by increased oxidative stress and apoptosis (Matzinger et al., 2018; Sireesh et al., 2018). In turn, pharmacologically activating Nrf2 reversed the high glucose-induced migration of damaged keratin-forming cells and promoted extracellular matrix deposition, accelerating wound closure (Long et al., 2016) (Jindam et al., 2017). It is also found that PZH could inhibit mitochondrial ROS-mediated neuronal apoptosis in ischemic stroke neuronal cells (Zhang X. et al., 2018) and protect liver function from damage by attenuating oxidative stress, inflammation, and mitochondrial apoptosis through the p53 signaling pathway (Zhao et al., 2017). These studies suggest that PZH may play an essential role in antioxidative stress and cytoprotection. However, the link between Nrf2/ARE pathway and PZH effective function is uncertain.

In this study, we evaluated the therapeutic potential of topical application and oral gavage of PZH in different species and different types of STZ-induced diabetic rats and/or mouse wounds models and explored whether the possible mechanism is based on the activation of Nrf2 and its downstream pathway to increase the expression of antioxidant enzymes and reduce ROS production, which can protect cell function from damage in human umbilical vein endothelial cells (HUVECs) under oxidative stress.

## 2 Materials and methods

### 2.1 Animals

Male Sprague‒Dawley (SD) rats (8–10 weeks, 350 ± 50 g) and male C57BL/6 mice (10–12 weeks, 20 ± 5 g) were purchased from Hunan SJA Experimental Animal Co Ltd. (Changsha, Hunan, China). The rats and mice were housed in pathogen-free conditions in the Department of Laboratory Animals, Central South University. All animal care, surgical procedures, and postoperative interventions were performed by the Guide for the Care and Use of Laboratory Animals. All animal studies were approved by the Animal Management Committee of Central South University (CSU-2022-0573).

### 2.2 Drugs

PZH was obtained from and certified by Zhangzhou PZH Pharmaceutical Company Limited (Zhangzhou, China; Chinese FDA approval No. Z35020242). Its chemical profile has been analyzed through HPLC or UPLC in many studies which have shown similar results that PZH was composited by ginsenoside Rg1, ginsenoside Rg2, ginsenoside Rg3, notoginsenoside R1, ginsenoside Re, ginsenoside Rf, ginsenoside Rh1, ginsenoside Rb1, ginsenoside Rd, glycocholic acid, taurochenodeoxycholic acid, tauroursodeoxycholic acid, taurocholic acid, cholic acid, ursodeoxycholic acid, hyodeoxycholic acid, glycodeoxycholic acid, chenodeoxycholic acid, deoxycholic acid, taurine and muscone (Qiu et al., 2018) (Zhang et al., 2022; Zhang C. et al., 2018; Huang et al., 2016).

### 2.3 Creation of a diabetic wound model

#### 2.3.1 Establishment of the diabetic model

All animals were acclimatized and fed for 1 week. Then the rats were injected intraperitoneally with 35 mg/kg body weight of STZ (Biofroxx, Germany) (100 mM in citrate buffer at pH 4.5) to construct a type 1 diabetes model (Yue et al., 2020). Mice were fed an acclimatized diet followed by a high-fat diet (Research Diets D12492, United States) for 8 weeks and then injected intraperitoneally with 35 mg/kg body weight of STZ (100 mM in citrate buffer at pH 4.5) for five consecutive days to construct a type 2 diabetic mouse model (Zhang C. et al., 2018; Li et al., 2020). Induction of diabetes was confirmed by measuring random blood glucose values > 16.7 mmol/L on the third and seventh days after STZ injection using a blood glucose meter (Roche, Germany). Two weeks after injection, blood glucose was tested again to exclude animals with substandard blood glucose.

#### 2.3.2 Establishment of the wound model

Animals that met the blood glucose standard were fed for another month to the next step of wound model construction. Diabetic rats were anesthetized with 5% isoflurane (RWD, Shenzhen, China) and maintained with 2% isoflurane; diabetic mice were anesthetized with 3% isoflurane and maintained with 1% isoflurane, their backs were shaved and disinfected, and full circular wounds of 1.0 cm in diameter were created on the skin 2.0 cm away from the spine on the back of the rats and 0.6 cm in diameter on the skin in the middle of the back of the mice.

### 2.4 Animal grouping and treatment

#### 2.4.1 Type 1 diabetic rats

PZH topical treatment group used their own control, with ten rats total. The diabetic topical control group received regular dressing treatment and dressing changes for the wound. For the PZH topical treatment group, PZH powder was added to the same quality of distilled water to make its mass fraction 50% and mixed into a paste, which was applied topically according to the wound size, approximately 0.05 g/cm^2^. The wound was treated with a regular medical dressing and dressing change and was observed for 10 days.

#### 2.4.2 Mice with type 2 diabetes mellitus

The mice were grouped according to their blood glucose levels and body weight and then divided into a diabetic gavage control group and a PZH gavage treatment group. The diabetic gavage control group was gavaged with a solvent of 0.1% sodium carboxymethyl cellulose (CMCNa) (Jinkelong, Beijing, China), and the wound was treated with a regular dressing and dressing change. The PZH gavage treatment group used a 0.234 g/kg (equal to the clinal dose for adults) body weight dose of PZH per day for gavage (Qiu et al., 2018; Deng et al., 2020). The gavage dose was approximately 200 μl, and the wound surface was treated with a regular medical dressing. Ten days of continuous treatment were observed.

### 2.5 Wound healing assessment

The wounds of rats and mice were recorded with a camera on postoperative Days 0, 2, 4, 6, 8, and 10. The wound area was measured by ImageJ software to observe healing and calculate the wound healing rate: (%) = (1 - unhealed wound area/original area) x 100%.

### 2.6 Histological and immunohistochemical analysis

On the eighth and 10th days of wound repair in diabetic rats and mice respectively, after exposure to CO_2_ for 10 min in an airtight chamber, the animals were euthanized with 5% isoflurane. The wound bed and surrounding healthy tissue, which included the epidermis and dermis, were immediately removed and divided into three parts: one part was fixed in 4% paraformaldehyde at 4°C, and the rest was used for protein and RNA extraction. Specimens were sectioned and stained with hematoxylin and eosin (H&E) (Servicebio, Wuhan, China) for microscopic analysis of epithelialization. Angiogenesis was assessed by immunohistochemistry (IHC) analysis of endothelial cell marker CD31 expression, and sections were incubated with primary CD31 antibody (GB113151, Servicebio, Wuhan, China, dilution 1:1000) at 4°C overnight, followed by horseradish peroxidase (HRP-) conjugated goat anti-rabbit secondary antibody (GB23303, Servicebio, Wuhan, China, dilution 1:200). Immunoreactivity was detected with a 3,3-diaminobenzidine (DAB) kit (Servicebio, Wuhan, China). Photographs were taken at ×100 and ×200 magnification using a microscope (Nikon DS-U3, Japan) and analyzed positive fractions using ImageJ.

### 2.7 Enzyme-linked immunosorbent assay

On the 10th day of wound repair in diabetic mice, after they were anesthetized with 3%isoflurane, their eyes were removed, and blood was collected from the mice. After standing for 3 h at room temperature, mouse serum was collected from the supernatant by centrifugation at 12000 rpm for 20 min at 4°C. Mouse serum IL-1β, IL-6, and TNF-α were assayed using ELISA kits (FANKEW, Shanghai, China) according to the manufacturer’s instructions.

### 2.8 Cell culture and treatment

HUVECs were cultured in 1640 medium (Procell, Wuhan, China), supplemented with 10% fetal bovine serum (Gibco, Life Technologies, Mulgrave, VIC, Australia), and incubated at 37°C in a humid air incubator with 5% CO2. PZH powder was well ground by the grinder and dissolved in physiological saline followed by sonication for 30 min (Li L. et al., 2018), then filtered through a 0.22 μm sterile filter (Millipore, Massachusetts, United States) to prepare 50 mg/ml PZH liquor. The resulting solution was used for cell experiments. Palmitic acid (PAL) (Solarbio, Beijing, China) was added to 1640 medium to a final concentration of 250 μM. Then, the configured master batch of PZH was added to a final concentration of 0.1 mg/ml, and HUVECs were treated for different durations.

### 2.9 Wound scratch migration assay

HUVECs were seeded in six-well plates at a density of 1 × 10^5^ cells/well and allowed to form a confluent monolayer. Each well was gently scratched in a straight line using a 10 μL pipette tip. Plates were washed with PBS to remove floating cells and then treated with 50 μM PAL and/or 0.1 mg/ml PZH containing 1% FBS and incubated at 37°C for 24 h. The cells were photographed immediately and 24 h after scratch using a normal optical microscope (100x) (Leica MC170HD, Germany). The migration of cells to the scratch bed was quantified using ImageJ software.

### 2.10 Tube formation assay

The tube formation assay was performed according to the manufacturer’s protocols of matrigel matrix (354234, Corning, NY, United States). The matrigel matrix was fully dissolved at 4 °C overnight for preparation. The matrigel matrix was added to each well of 96-well plates for 60 μL and incubated at 37 °C for 45 min. Then, cell culture medium containing 1 × 10^4^ HUVECs was seeded on the matrigel with or without 250 μM PAL and or 0.1 mg/mL PZH for 1.5 h. After 6 h, the tube formation of HUVECs was observed and photographed using a normal optical microscope (40x) (Leica MC170HD, Germany). The tube formation ability in each well was calculated by ImageJ software.

### 2.11 Measurement of intracellular ROS

Intracellular ROS levels were determined using ROS Assay Kit (Beyotime, Haimen, China) according to the manufacturer’s instructions. The cell was seeded in 6-well plate and incubated at 37°C until cell density reached 90%, then treated with 250 μM PAL and/or 0.1 mg/ml PZH for 3 h. Plates were washed twice with PBS and then added 10 μM 2,7-dichlorodihydrofluorescein diacetate (DCFH-DA) and incubated at 37°C for 15 min. The fluorescence intensity was monitored with an excitation wavelength of 488 nm and emission wavelength of 530 nm and photographed using a fluorescent microscope (Leica MC170HD, Germany). The level of intracellular ROS was expressed as the mean fluorescence intensity. Data were analyzed using ImageJ.

### 2.12 siRNA transfection

NRF2 gene expression was knocked down by the transfection of HUVECs with siRNA-NRF2 or siRNA-NC, which were designed and synthesized by Qingke Biotechnology Company (Beijing, China). Transient siRNA transfections were performed using Lipofectamine 3000 (Invitrogen, Carlsbad, CA, United States) according to the manufacturer’s instructions. After 60 h of transfection, HUVECs were treated with 250 μM PAL and/or 0.1 mg/ml PZH for 12 h. Lipofectamine solution and transfection efficiency were confirmed by western blotting.

The sense sequence:

Nrf2 siRNA sense 5′-GAG​UAA​GUC​GAG​AAG​UAU​UTT-3 antisense 5′- 5′-AAU​ACU​UCU​CGA​CUU​ACU​CTT-3'; Control siRNA sense 5′-UUC​UCC​GAA​CGU​GUC​ACG​UTT-3′ antisense 5′-ACG​UGA​CAC​GUU​CGG​AGA​ATT-3′

### 2.13 Cell proliferation assay

The cells were taken in a logarithmic growth phase, digested to make a single cell suspension for the count, and inoculated in 96-well plates at a density of 3000 cells/well. Marginal wells were sealed with PBS solution and incubated at 37°C for 24 h. Cell viability was determined by analysis using a CCK-8 kit (Apexbio, Houston, Texas, United States) according to the manufacturer’s instructions.

### 2.14 Western blotting

Cells and wound tissue were lysed in RIPA lysis buffer containing phenylmethanesulfonyl fluoride (biosharp, Guangzhou, China). Lysates were separated by centrifugation at 12,000 × g for 10 min at 4 °C, and the supernatant was subjected to sodium dodecyl sulfate-polyacrylamide gel electrophoresis (SDS‒PAGE). The proteins were transferred onto polyvinylidene fluoride (PVDF) membranes (ISEQ00010, Millipore, Massachusetts, United States). After being closed in TBST containing 5% skimmed milk for 1 h at room temperature, the membranes were incubated overnight at 4°C with primary antibodies to c-Jun (24909-1-AP, Proteintech, Wuhan, China, dilution 1:1000), VEGF (19003-1-AP, Proteintech, dilution 1:1000), EGF (DF2225, Affinity, Shanghai, China, dilution 1:250), PDGF (ab178409, Abcam, Cambridge, United States, dilution 1:1000), Nrf2 (16396-1-AP, Proteintech, dilution 1:1000), HO-1 (R22808, Zen Bio, Chengdu, China, dilution 1:1000), SOD-1 (10269-1-AP, Proteintech, dilution 1:1000), p53 (60283-2-Ig, Proteintech, dilution 1:1000), Bax (50599-2-Ig, Proteintech, dilution 1:1000), cleaved caspase-3 (19677-1-AP, Proteintech, dilution 1:1000), GAPDH (10494-1-AP, Proteintech, dilution 1:1000), and β-tubulin (AP0064, Bioworld, MN, United States, dilution 1:1000), respectively. The membranes were then incubated with HRP-conjugated secondary antibodies (511103, 511203, Zen Bio, dilution 1:5000), and the protein bands were detected by chemiluminescence (BioRad, United States).

### 2.15 Real-time Reverse Transcription polymerase chain reaction

Total RNA was extracted from wound tissue using TRIzol reagent (CWBio, Jiangsu, China). cDNA was synthesized using the RT Reverse Transcription kit (Yeasen, Shanghai, China). qPCR was performed using the SYBR Green Real-Time PCR Master Mix kit (Yeasen, Shanghai, China) under the following conditions: predenaturation at 95°C for 5 min, at 95°C for 15 s, at 58°C for 20 s and at 72°C for 20 s for 40 cycles (Roche LightCycle 480 Ⅱ, Germany). Relative gene expression was calculated using the 2^−ΔΔCt^ formula, and the data were normalized to β-actin.

PCR primer sequences were as follows: β-actin, 5′-CTA​CCT​CAT​GAA​GAT​CCT​CAC​C-3′ (forward) and 5′- AGT​TGA​AGG​TAG​TTT​CGT​GGA​T-3′ (reverse); c-Jun, 5′-GCC​TAC​AGA​TGA​ACT​CTT​TCT​GGC-3’ (forward) and 5′-CCT​GAA​ACA​TCG​CAC​TAT​CCT​TTG-3’ (reverse); VEGF-A, 5′-GTG​ACA​AGC​CAA​GGC​GGT​GAG-3′ (forward) and 5′-GAT​GGT​GGT​GTG​GTG​GTG​ACA​TG-3′ (reverse); PDGF, 5′-TGA​ATC​TCC​CTC​GCA​GAG​CA-3′ (forward) and 5′-CTC​ATC​GCC​ACC​TTG​ACA​CC-3′ (reverse).

### 2.16 Statistical analysis

Data were analyzed using GraphPad Prism 7.0 software. All data are expressed as the mean ± standard deviation (SD), and differences between the two groups were analyzed using an unpaired Student t-test. One-way analysis of variance (ANOVA) was used to explain the differences between the multiple groups, *p* < 0.05 was considered statistically significant.

## 3 Results

### 3.1 Administration of PZH promotes wound healing in diabetic rats/mice

**FIGURE 1:**
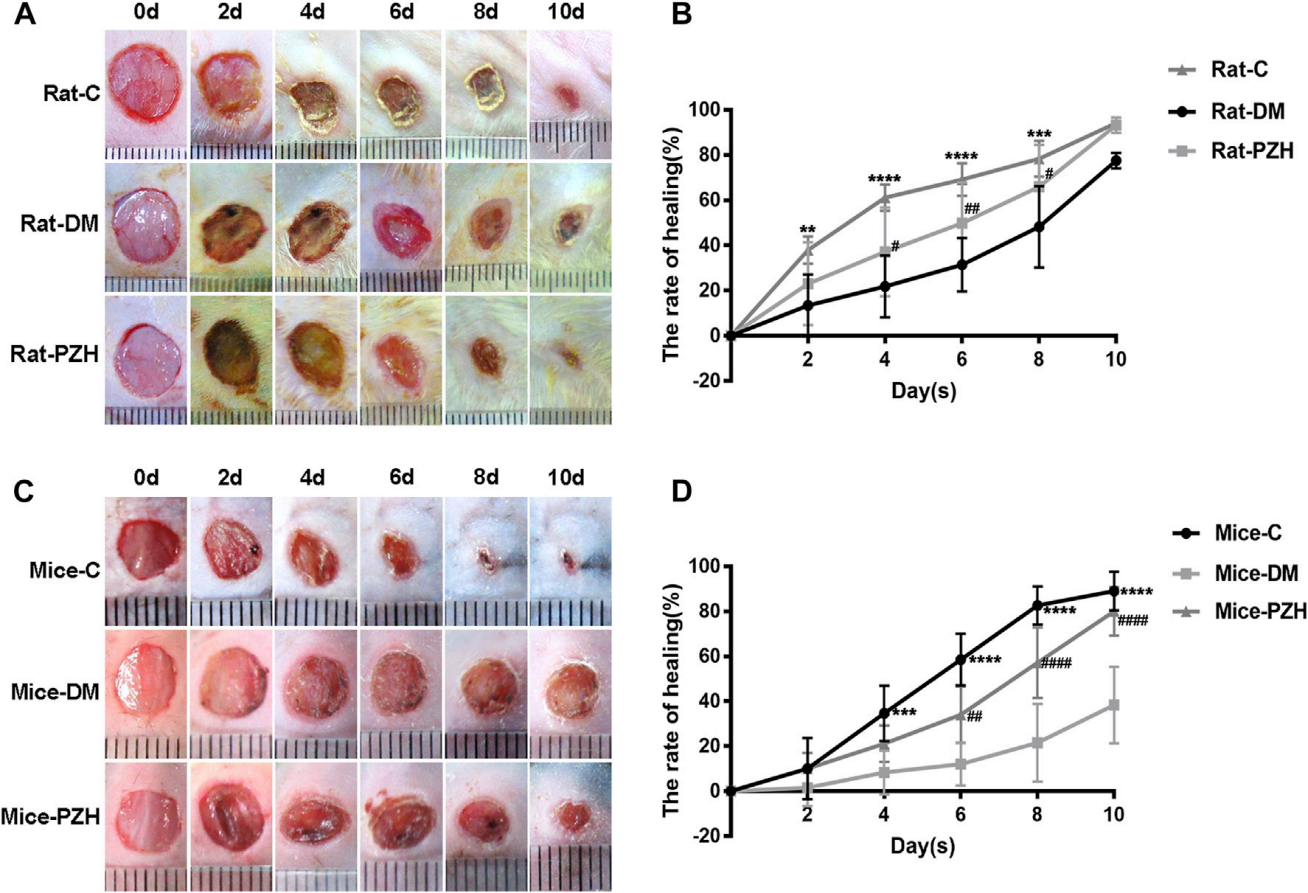
PZH promotes wound healing in diabetes models. **(A)** Representative photos of the wound healing process in different groups of rats. **(B)** Wound healing rate of different groups of rats. Data are expressed as the mean ± SD. C vs. DM: ^**^ *p* < 0.01, ^***^ *p* < 0.001, ^****^ *p* < 0.0001 (n = 4); PZH vs. DM: ^#^ *p* < 0.05, ^##^ *p* < 0.01 (n = 9). **(C)** Representative photos of the wound healing process in different groups of mice. **(D)** Wound healing rate of different groups of mice. C vs. DM: ^***^ *p* < 0.001, ^****^ *p* < 0.0001 (n = 6); PZH vs. DM: ^##^ *p* < 0.05, ^####^ *p* < 0.0001 (n = 6). C: normal control group, DM: diabetes model control group, PZH: diabetes model with PZH treatment group.

All the diabetic models were sure to meet the standard blood glucose (>16.7 mmol/L) (Supplementary Figure 1). Next, we established a full-layer excision wound on the back skin of diabetic rats and mice and checked the wound closure every 2 days. Figure 1 shows easier wound closure and a higher healing rate in the normal control group than in the diabetic group, suggesting that the diabetic wound model was successfully established. In type 1 diabetic rats, topical treatment with PZH improved the wound healing ability (Figure 1A). The wound healing rate was significantly faster as early as the fourth day of wound repair in the topical PZH-treated wounds compared to the diabetic control group (Figure 1B). Similarly, wound healing in type 2 diabetic mice was also improved after gavage treatment with PZH, and a significant enhancement of the wound healing rate could be observed from Day 6 to Day 10 in wounds treated by PZH gavage compared to wounds treated by CMCNa gavage (Figures 1C, D). These results indicated that both topical treatment and oral gavage treatment with PZH could improve the healing of diabetic wounds and play a positive role in diabetic wound repair. In addition, we found that the effect on blood glucose was insignificant after PZH gavage treatment (Supplementary Figure 1E).

### 3.2 Histological evaluation of wounds: PZH accelerated the Re-epithelialization and angiogenesis of wound tissue

**FIGURE 2:**
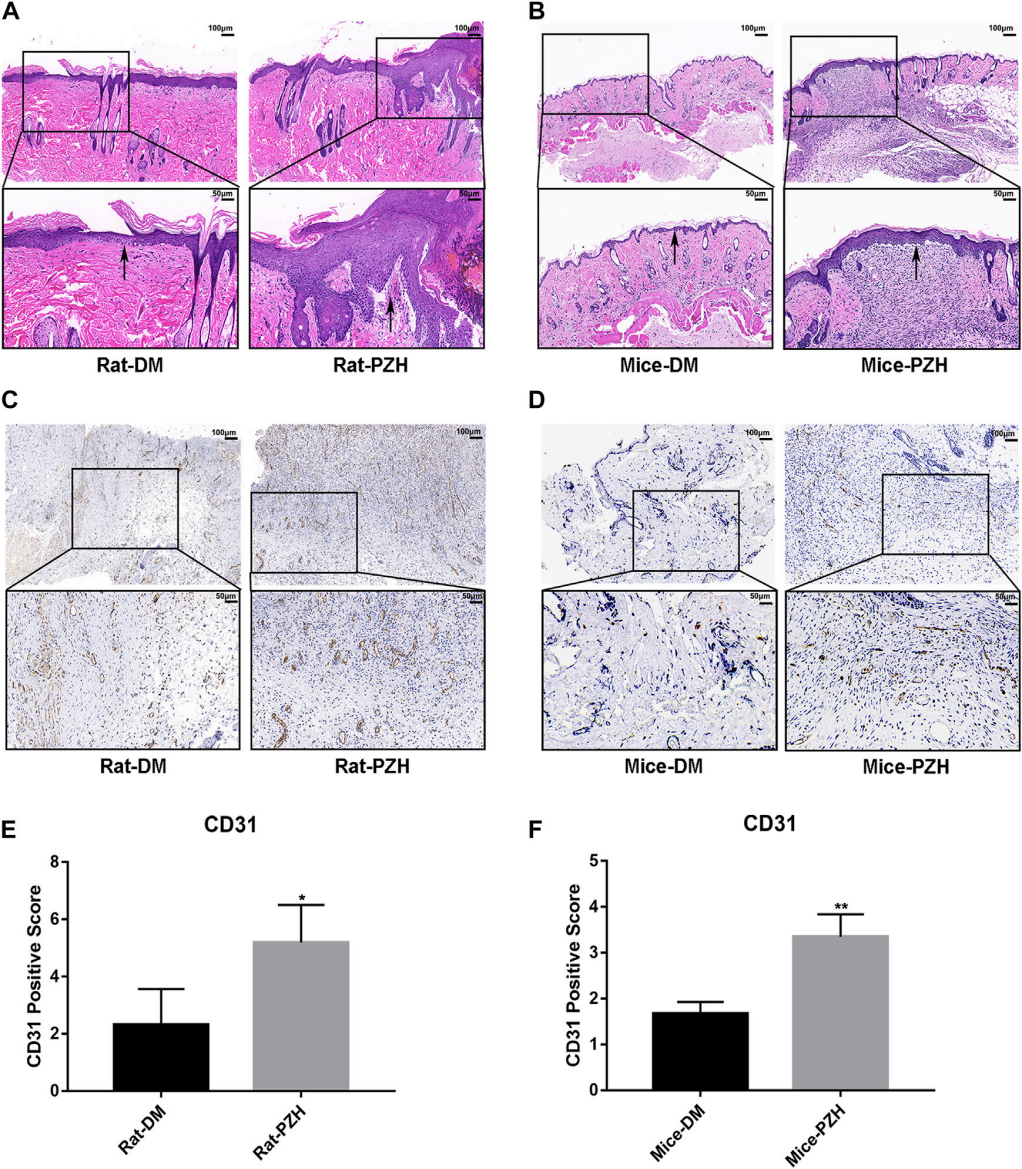
PZH accelerated the re-epithelialization and angiogenesis of diabetic rat/mice wounds. **(A,B)** On the eighth/10th day of topical/gavage treatment with PZH, images of H&E staining (scale bar = 100 μm, magnification ×100; scale bar = 50 μm, magnification ×200) of wound skin tissue sections of different groups of rats **(A)** or mice **(B)** were examined under a light microscope. Black arrow: epithelial layer. DM: diabetic control group; PZH: PZH-treated group. **(C–F)** Histological images of CD31 staining and quantitative analysis of wound tissue in different groups of rats **(C,E)** or mice **(D,F)** under a light microscope. Blood vessels were stained for CD31, and brown staining was positive for CD31. ^*^ *p* < 0.05 (n = 4), ^**^ *p* < 0.01 (n = 3). Data are expressed as the mean ± SD.

It is well known that restoration of normal skin tissue structure is an important indicator of wound healing. The H&E staining results (Figures 2A, B) showed that after topical or gavage treatment with PZH, the wound epithelium structure in diabetic rats and mice was clearer than that in the diabetic control groups, and the thickness of the epithelial layer was increased, suggesting that PZH treatment might have accelerated epithelialization to improve wound healing in diabetic rats and mice.

**FIGURE 3:**
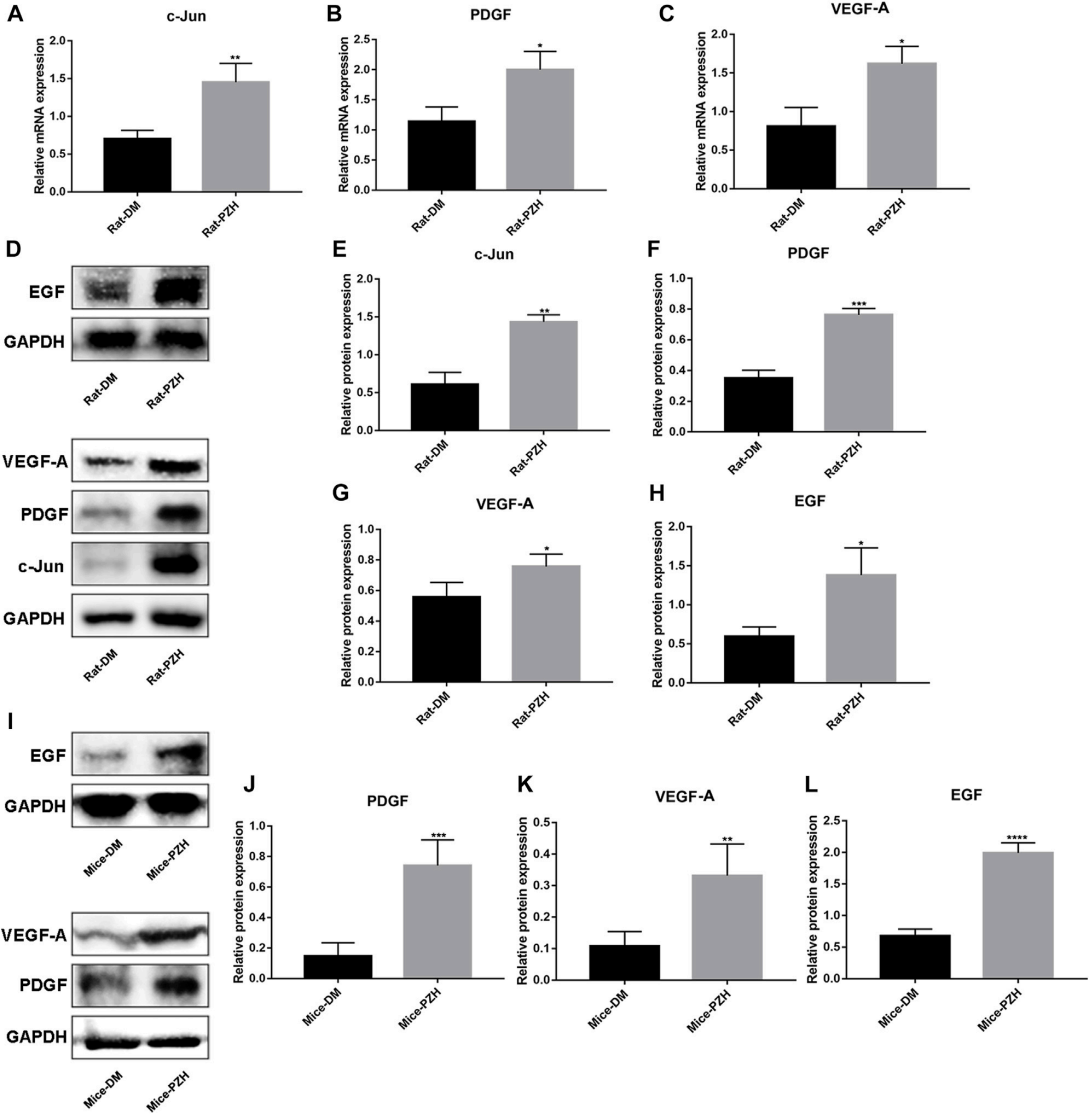
PZH increased the expression of growth factors in diabetic wounds. **(A–C)** Effect of topical treatment with PZH on the eighth day of wound healing at the mRNA levels of c-jun **(A)**, PDGF **(B)**, and VEGF-A **(C)** in diabetic rat wounds. ^*^ *p* < 0.05, ^**^ *p* < 0.01 (n = 3). **(D–H)** The protein level of c-jun, VEGF-A, PDGF, and EGF was detected in type 1 diabetic rat wounds on the eighth day of topical treatment with PZH, **(D)** is representative blotting, band intensity was quantified using ImageJ, right panels are quantification of c-jun **(E)**, PDGF **(F)**, VEGF-A **(G)** and EGF **(H)** level, ^*^ *p* < 0.05, ^**^ *p* < 0.01, ^***^ *p* < 0.001 (n = 3). (I–L) The protein level of PDGF **(J)**, VEGF-A **(K)**, and EGF **(L)** were detected in type 2 diabetic mouse wounds on the 10th day of PZH gavage treatment by Western blotting, and band intensity was quantified using ImageJ, **(I)** is representative blotting. ^*^ *p* < 0.05, ^**^ *p* < 0.01 (n = 4). Data are expressed as the mean ± SD. DM: diabetic control group; PZH: PZH-treated group.

Impaired angiogenesis is one of the obstacles to diabetic wound closure (Zins et al., 2010). We performed IHC staining of CD31 to evaluate capillary densities. The IHC staining of tissue sections revealed increased vasculature in wound tissues in the PZH-treated groups compared with the diabetic control groups in the two diabetes models (Figures 3C, D). Moreover, the quantitative calculation of positive staining for CD31 showed that the capillary density was significantly higher than that in the control groups (Figures 3E, F), indicating that treatment with PZH prompted angiogenesis in diabetic wounds.

### 3.3 Administration of PZH increases growth factor levels in wound tissue

Wound healing is a complex, highly regulated process that requires the interplay of multiple cell types and their secreted cytokines. Therefore, we detected the expression of some growth factors in the wound tissue after PZH treatment. On the eighth/10th day of wound repair in rats/mice, we performed RNA and/or protein extraction from the wound tissue. The results showed higher levels of c-jun, VEGF-A, and PDGF mRNA and protein expression, as well as EGF protein expression, in the rat wound tissue of the diabetic PZH-treatment group compared to the diabetic control group (Figures 3A–H). Similarly, we observed that the protein expression levels of EGF, PDGF, and VEGF-A were increased in the wounds of diabetic mice after PZH gavage treatment (Figure 3I-L).

### 3.4 PZH induces activation of the Nrf2/ARE pathway in wound tissue and reduction of inflammatory factors in blood

**FIGURE 4:**
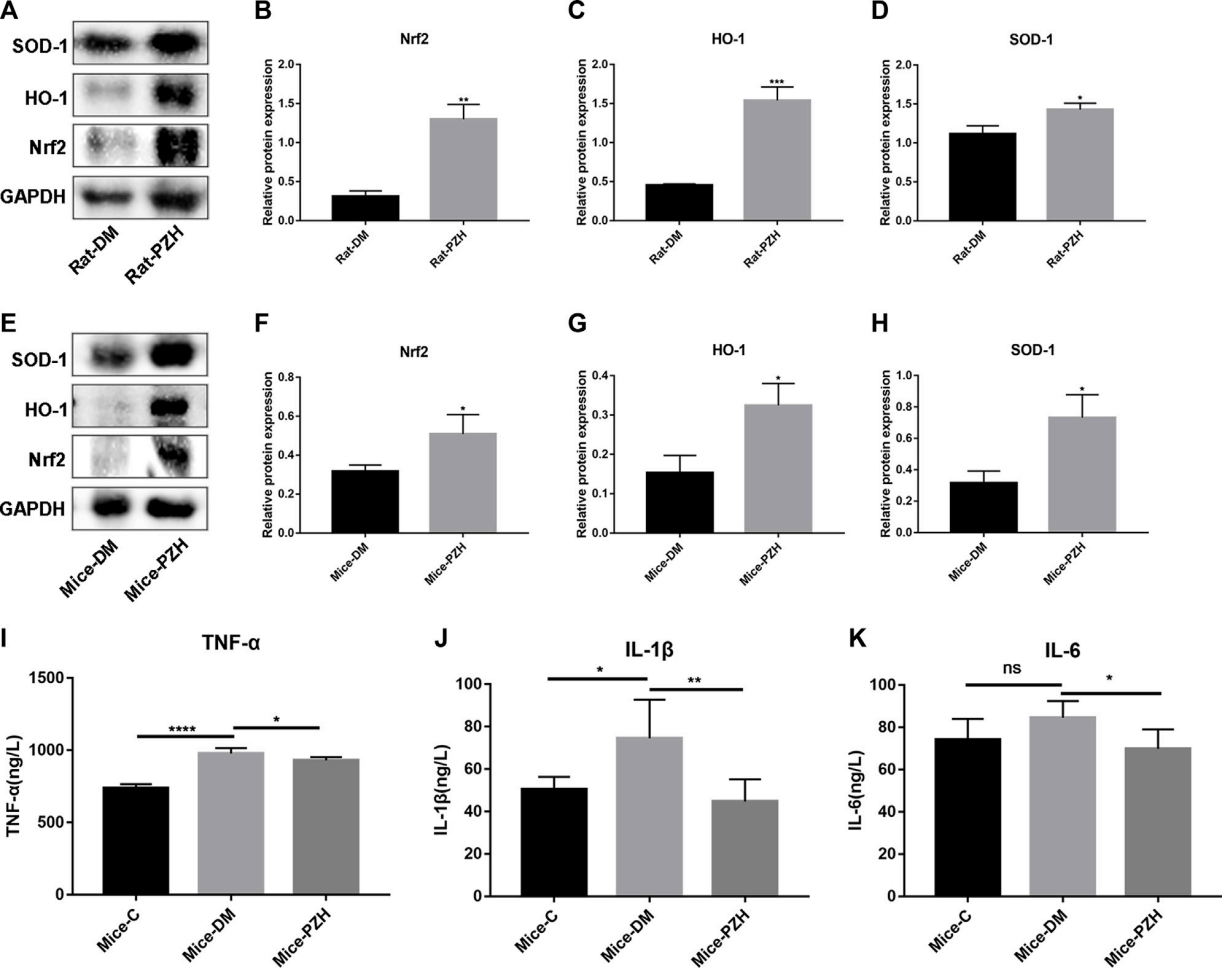
PZH activates the Nrf2/ARE pathway in diabetic wound tissue and reduces serum inflammatory factors in diabetic mice. **(A–H)** Western blotting and quantification of Nrf2, HO-1, and SOD-1 in the wound tissue of diabetic rats (A–D) or mice (E–H). ^*^ *p* < 0.05, ^**^ *p* < 0.01, ^***^ *p* < 0.001 (n = 3). (I–K) ELISA detection of serum inflammatory factor levels in different groups of mice: TNF-α **(I)**, IL-1β **(J)**, IL-6 **(K)**. ^****^ *p* < 0.0001, ^**^ *p* < 0.01, ^*^ *p* < 0.05 (n = 5). Data are expressed as the mean ± SD. C: normal control group; DM: diabetic control group; PZH: PZH-treated group.

Since oxidative stress has acted as a crucial factor in chronic non-healing wounds in diabetes (Kant et al., 2014) (Newsholme et al., 2016), and PZH has been shown in some studies to reduce mitochondrial ROS production and attenuate oxidative stress, while Nrf2 is a key responder to stress transcription factor concerned with the cellular response to oxidative stress (Loboda et al., 2016), in order to explore the underlying mechanism of PZH effective effects, we measured the level of the NRF2/ARE pathway in the wound tissue after PZH treatment. The western blotting results showed that the protein expression levels of Nrf2 and its downstream targets HO-1 and SOD-1 were upregulated in the wound tissue of the diabetes models treated with topical or intragastric PZH compared with the control groups. (Figures 4A–H). This finding suggests that PZH treatment induces activation of the Nrf2/ARE pathway in wound tissue.

In addition, PZH is traditionally used to treat various inflammatory diseases and reduce wound swelling and pain. Therefore, we collected serum from mice on the 10th day after gavage to detect the levels of inflammatory factors such as IL-1β, IL-6, and TNF-α. The ELISA results illustrated that the serum levels of all three proinflammatory cytokines in the diabetic group were higher than those in the normal control group (Figures 4I–K), while they were suppressed after gavage treatment with PZH.

### 3.5 PZH protects HUVECs from PAL-induced oxidative impairment

**FIGURE 5:**
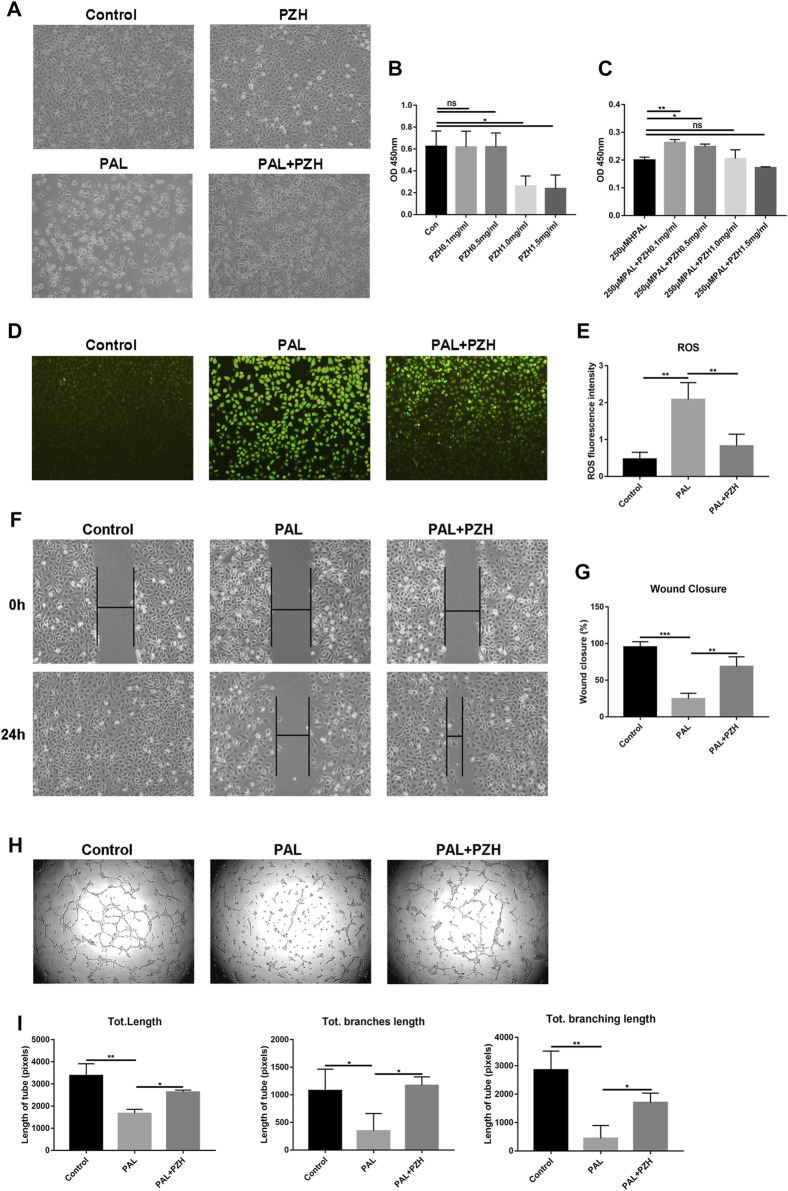
PZH protects HUVECs from impaired function in PAL. **(A)** The effects of PZH (0.1 mg/mL) on the cell growth status of HUVECs after exposure to PAL (250 μM) for 24 h **(B,C)** The proliferation was detected by CCK8 kits in HUVECs at different concentrations of PZH intervention for 24 h without **(B)** or with 250 μM PAL intervention **(C)**. ^*^ *p* < 0.05, ^**^ *p* < 0.01 (n = 3). **(D,E)** The effects of PZH on antioxidative stress in HUVECs after exposure to PAL **(D)** by using DCFH-DA staining to detect the ROS level, the fluorescent intensity **(E)** was quantified by ImageJ ^**^ *p* < 0.01, (n = 3). **(F–G)** The effects of PZH on HUVECs migration **(F)** was assessed at 0 and 24 h following cell-scratching. The wound closure rate **(G)** was quantified by ImageJ. ^**^ *p* < 0.01, ^***^ *p* < 0.001 (n = 3). **(H–I)** The effects of PZH on the angiogenic function **(H)** of HUVECs were determined by tube formation assay. The tube length **(I)** was quantified by ImageJ. ^*^ *p* < 0.05, ^**^ *p* < 0.01 (n = 3).

The above data suggested that PZH could alleviate oxidative stress by activating the Nrf2/ARE pathway to promote healing. As oxidative stress and the increase of ROS are the important mechanisms of endothelial dysfunction and ischemic neovascularization disorder (Dehghani et al., 2020), which are involved in wound healing in diabetes. So we constructed an oxidative stress model by stimulating HUVECs with PAL to observe the effect of PZH (0.1, 0.5, 1.0, and 1.5 mg/ml) on the function of HUVECs under oxidative stress conditions (Batumalaie et al., 2016; Xing et al., 2019). We observed that HUVECs treated with PZH had better cell growth status than HUVECs cultured with PAL control. The cell proliferation assessed by CCK8, it showed that the proliferation ability of HUVECs cultured in PAL was significantly improved under PZH treatment with concentrations of 0.1,0.5 mg/mL, while the proliferation ability of HUVECs cultured under normal conditions was unchanged (Figures 5A–C). In addition, PZH concentrations over 1.0 mg/ml significantly inhibited cell proliferation. Meanwhile, the migration and tube formation abilities of HUVECs were evaluated by wound scratch assay and matrigel tube formation assay respectively. The results showed that PZH treatment improved inhibited migration and tube formation of HUVECs cultured in PAL (Figures 5F–I). Next, we detected the ROS level, PAL-induced high ROS level in HUVECs was remarkably reduced by PZH treatment (Figures 5D, E). These results suggested that PZH at an appropriate concentration may protect vascular endothelial cells from oxidative stress-induced damage.

### 3.6 PZH attenuates PAL-induced oxidative stress and apoptosis in HUVECs *via* activating Nrf2/ARE pathway

**FIGURE 6:**
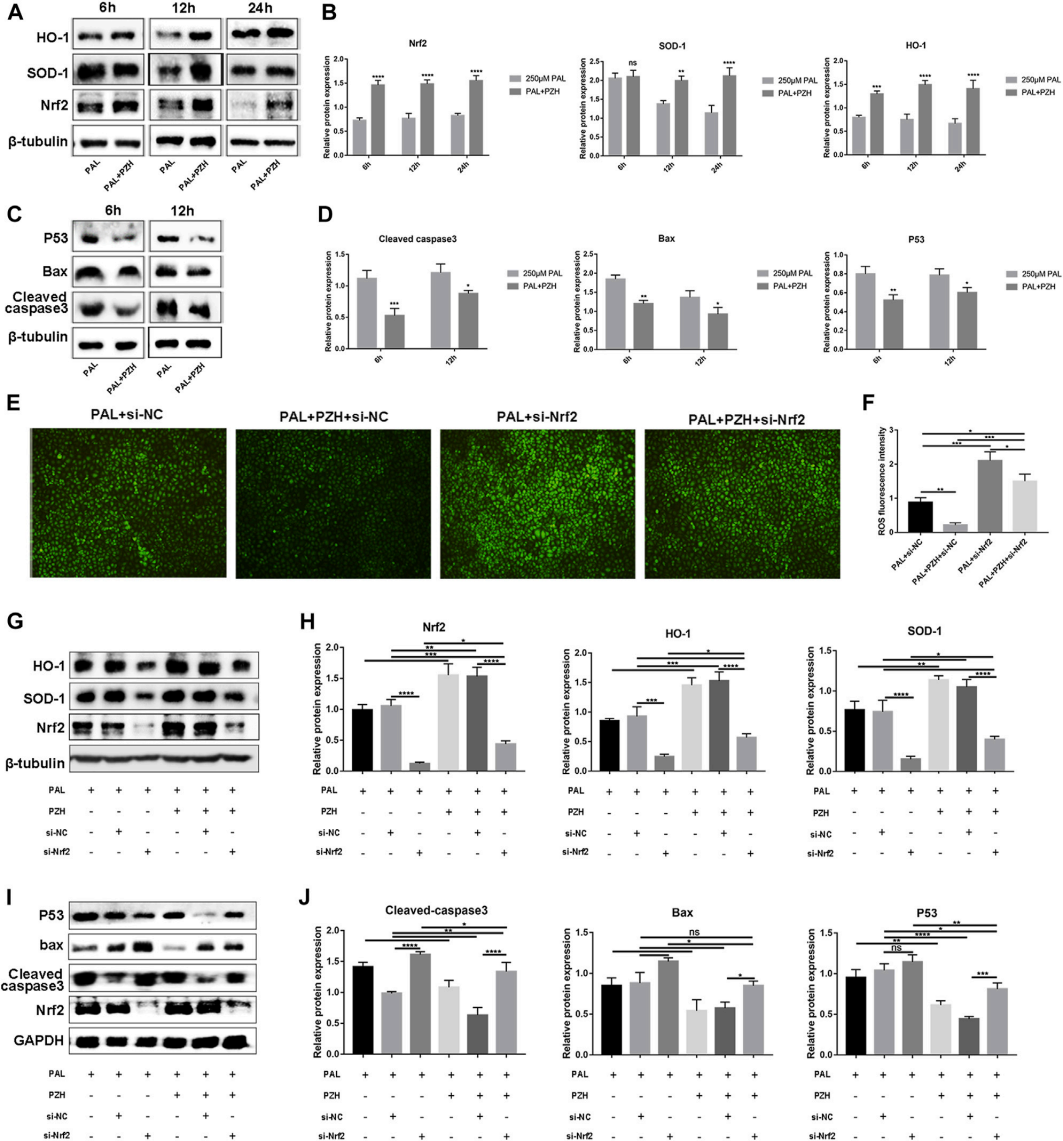
PZH attenuates PAL-induced oxidative stress and apoptosis in HUVECs *via* activating the Nrf2/ARE pathway. Knockdown of Nrf2 abolishes the anti-oxidative, and anti-apoptosis effects of PZH on HUVECs treated with PAL. **(A–F)** Effect of 0.1 mg/ml PZH on the expression and quantitative analysis using ImageJ of Nrf2 pathway-related proteins **(A,B)** and apoptotic proteins **(C,D)** at different hours in HUVECs treated with 250 μM PAL. ^****^ *p* < 0.0001, ^***^ *p* < 0.001, ^**^ *p* < 0.01, ^*^ *p* < 0.05 (n = 3). **(E,F)** The effects of Nrf2 knockdown on the anti-oxidative stress of PZH in HUVECs were determined by DCFH-DA staining to detect the ROS level **(E)**, the fluorescent intensity **(F)** was quantified by ImageJ ^*^ *p* < 0.05, ^**^ *p* < 0.01, ^***^ *p* < 0.001, (n = 3). **(G,H)** Protein expression and quantitative analysis using ImageJ of Nrf2/HO-1 and SOD-1 in HUVECs treated with Nrf2-siRNA. ^*^ *p* < 0.05, ^**^ *p* < 0.01, ^***^ *p* < 0.001, ^****^ *p* < 0.0001 (n = 3). **(I,J)** Protein expression and quantitative analysis of Bax, p53, and cleaved caspase-3 induced by 0.1 mg/ml PZH in HUVECs cultured in 250 μM PAL and treated with Nrf2-siRNA. ^*^ *p* < 0.05, ^**^ *p* < 0.01, ^***^ *p* < 0.001, ^****^ *p* < 0.0001 (n = 3). Data are expressed as the mean ± SD.

We then tested the expression of Nrf2 and its downstream proteins HO-1 and SOD-1 in HUVECs cultured in PAL at 6, 12, and 24 h after PZH treatment. The expression of p53, Bax and cleaved caspase-3 was decreased after intervention with 0.1 mg/ml PZH at the same time (Figures 6A–D). The results revealed that the expression of Nrf2 and HO-1 in HUVECs was significantly increased as early as 6 h, while the expression of SOD-1 was increased at 12 h and 24 h (Figures 5A, B). These results demonstrate that PZH may be associated with activating the Nrf2/ARE pathway.

To investigate whether PZH mediates the protective effect of PAL on HUVECs cultured by the Nrf2/ARE pathway, Nrf2 expression was knocked down with Nrf2-siRNA in subsequent experiments. We found that knockdown of Nrf2 could not only abolishes the antioxidative effects of PZH on HUVECs treated with PAL, but also attenuated PZH-induced HO-1 and SOD-1 expression and inhibited PZH-induced downregulation of Bax, p53, and cleaved caspase-3 in HUVECs cultured in PAL (Figures 6E–J). The above results indicate that PZH protects HUVECs from an oxidative stress-induced impairment, at least in part, by activating the Nrf2/ARE pathway partly, thus boosting diabetic wound healing.

## 4 Discussion

PZH is a traditional Chinese remedy that has been found to be effective in treating inflammatory diseases and cancer, more recent studies have investigated its pharmacological effects (Chen, 2021). In our research, we observed for the first time that PZH facilitates wound healing in different biological diabetic models, promotes the expression of NRF2 and its downstream antioxidant genes HO-1 and SOD-1, increases the expression of VEGF-A, PDGF and EGF in diabetic wounds, and reduces the levels of TNF-α, IL-1β, and IL-6 in diabetic mouse serum, contributing to the repair of diabetic wounds. *In vitro* assay, it was demonstrated that PZH could against excessive oxidative stress and protect HUVECs from PAL-induced oxidative impairment by activating Nrf2/ARE pathway.

In diabetic patients, there are multiple mechanisms for delayed wound healing, including impaired expression of growth factors and angiogenesis (Dehghani et al., 2020), a prolonged inflammatory phase (Zubair and Ahmad, 2019) as well as excessive oxidative stress, which can induce a series of cascade responses in the body, increases cell apoptosis and senescence by downregulating cytoprotective mechanisms (Schäfer and Werner, 2008) further leading to delayed diabetic wound healing (Avila et al., 2013; Eming et al., 2014). Thus, improving the oxidative stress state and persistent inflammation, increasing wound tissue growth factors, as well as stimulating the potential for angiogenesis are the critical starting points for wound healing (Kant et al., 2014; Cano Sanchez et al., 2018). Nrf2 is an essential transcriptional regulator of genes, which controls the adaptive response to oxidative stress, reduces apoptosis, and promotes cell migration, proliferation, and differentiation, thus exerting antitumor, anti-inflammatory, and anti-apoptotic effects (Niture and Jaiswal, 2014; Matzinger et al., 2018) (Tonelli et al., 2018). In particular, heme oxygenase 1 (HO-1) is one of the key downstream target genes regulated by Nrf2. It is involved in regulating critical biological processes, including inflammation, apoptosis, cell proliferation, fibrosis, and angiogenesis (Loboda et al., 2016). All of these vital physiological regulatory processes are inextricably linked to diabetic wound healing.

Several studies have confirmed that Nrf2/ARE pathway plays a pivotal role in facilitating wound repair (Li et al., 2019; Wang et al., 2020), and deficiencies in either SOD-1 or HO-1 delayed the wound healing process in mice (Deshane et al., 2007; Iuchi et al., 2010). In our research, PZH facilitates diabetic wound healing, the expression of Nrf2, HO-1, and SOD-1 were upregulated in the wound tissue of the diabetes models treated with topical or intragastric PZH. *In vitro* assay, it is shown that PZH decreased PAL-induced high ROS levels in HUVECs, improved the proliferation, migration, and tube formation abilities of HUVECs cultured in PAL, reduced the expression of cleaved-caspase3, p53, and these protective effects were abrogated by specific Nrf2 siRNA. These data indicate that PZH promotes wound healing in diabetes models associated with activating Nrf2/ARE Pathway.

Excluding the mediation of redox reactions, Nrf2 signaling also suppresses inflammation by inhibiting proinflammatory cytokines (Ahmed et al., 2017). Nrf2 can negatively regulate NLRP3 inflammasome activity and the NF-kB pathway by inhibiting ROS-induced activation (Liu et al., 2017). Our data showed that PZH treatment was able to reduce TNF-α, IL-1β, and IL-6 levels in diabetic mouse serum, which had a positive effect on wound healing. Previous studies have shown that the anti-inflammatory effect of PZH is associated with the inhibition of the NLRP3 inflammasome and NF-kB pathway (Li L. et al., 2018; Deng et al., 2020; Huang et al., 2021; Lian et al., 2022). However, activation of NF-kB can inhibit Nrf2 activity (Vijayan et al., 2018). Since there is functional crosstalk between Nrf2 and NF-kB transcription factors, it is not difficult for us to surmise the connection between them. In addition, PZH may have multiple signal pathways related to anti-inflammatory mechanisms due to its various active components. Taken together, our study shows that PZH has an active role in both anti-inflammation and antioxidative stress, which contribute positively to diabetic wound repair. However, as for the anti-inflammatory effect of PZH, more inflammatory indicators, including the local anti-inflammatory effect need to be evaluated, and the anti-inflammatory mechanism needs to be further explored.

Insufficient angiogenesis plays a vital role in the pathogenesis of poor wound healing and micro and microvascular diseases in diabetes. Oxidative stress induced by glucolipotoxicity in diabetes patients is the root cause of many microvascular and macrovascular complications, which will eventually affect angiogenesis. Moreover, endothelial cell (EC) dysfunction is the initial and permanent factor for the development of vascular complications and angiogenesis disorders in diabetes (Kolluru et al., 2012) (Vlassara et al., 2002). Excessive oxidative stress and sustained inflammatory state is the most prevailing mechanism of endothelial dysfunction in diabetes. Therefore, in our vitro assays, we evaluated the effect of PZH on HUVEC function under oxidative stress conditions. The results showed that PZH at an appropriate concentration protects vascular endothelial cells from oxidative stress-induced damage, decreases susceptibility to apoptosis, and improves the angiogenic and migration ability of HUVECs under PAL-induced oxidative stress. Moreover, in our study, higher levels of VEGF-A, PDGF, and EGF protein expression, a thicker epithelial layer, and more vasculature marked by CD31 were also observed in wound tissue after PZH treatment, implying that PZH promotes angiogenesis, accelerates wound healing. Since its various active ingredients, PZH may promote angiogenesis through multiple signaling pathways. Nrf2 plays a critical role in angiogenesis which has been increasingly demonstrated, it triggers antioxidant pathways to exert cytoprotective effects in vascular endothelial cells or endothelial progenitor cells (Li X. et al., 2018; Chen et al., 2019). Hyperbaric oxygen therapy promoted wound healing by increasing levels of Nrf2, which was positively correlated with EGF, VEGF-A, and PDGF (Dhamodharan et al., 2019). In our research, it suggests that PZH promotes angiogenesis is at least partly associated with the activating Nrf2/ARE pathway in diabetic wound healing. Moreover, we also observed that the expression of c-jun, one of the transcription factors of the AP-1 family, which plays an essential role in cell proliferation and migration and is directly related to many processes in the skin, was increased in diabetic wounds treated with topical application of PZH (Angel and Szabowski, 2002; Yates and Rayner, 2002). Studies have shown that c-jun can bind to the promoter regions of the VEGF-A and PDGF genes to facilitate the transcription of VEGF-A and PDGF-B (Wei et al., 2002; Yin et al., 2009). This is consistent with our observation that the levels of c-jun, VEGF-A, and PDGF mRNA and protein in diabetic wounds treated with PZH were increased, suggesting that the ability of PZH to elevate the levels of growth factors in wounds may be due to increased expression of c-jun. However, due to the complex composition of PZH, we could not determine the exact mediation between growth factors and the Nrf2/ARE pathway or other signaling pathways, which needs to be further proven.

On the other hand, it seems to contradict our results that some research reported PZH was used in oncology treatment in association with its anti-angiogenic effects (Shen et al., 2013). It may be that the angiogenesis in the tumor microenvironment is different from the diabetic wound healing state, the process of angiogenesis is highly regulated by multiple cellular signal transduction pathways, and the complex interactions between molecules and how they are in different environments can affect the structure and function of blood vessels. The physiological expression of angiogenic factors is tightly regulated, in addition to tumor-induced sustained angiogenesis, which can be facilitated by mutations leading to increased expression of activated oncogenes or loss of tumor suppressor genes (Folkman and Klagsbrun, 1987). Angiogenesis can be initiated by responses to inflammation, hypoxia, and other conditions and is mediated by vascular regulatory factors released from tumors and other abnormal cells (Nyberg et al., 2005). For example, hypoxia can further stimulate angiogenesis in the tumor by activating the HIF pathway (Semenza, 2002), which proved to be inhibited by PZH in human colon carcinoma cells (Chen et al., 2014). In our study, we observed that PZH has an active role in both anti-inflammation and antioxidative stress, which can change the hypoxia state (McGarry et al., 2018), may be associated with the effects of preventing pathological angiogenesis in tumors but take good effects in diabetic wound angiogenesis to promote healing (Tu et al., 2022). However, the specific regulatory mechanism of PZH on angiogenesis is not clear. The functional roles of PZH in angiogenesis are complex and not fully understood. Further studies are needed to determine the use of PZH and to reveal the molecular mechanisms. In addition, in this study, the mechanism of PZH promoting angiogenesis involves Nrf2. Recent studies have found that Nrf2 has a dual role in promoting and inhibiting tumors. Nrf2 reduces cancer risk by inhibiting oxidative stress and tumor-promoting inflammation; On the other hand, it is a driving factor for cancer progression, metastasis, and treatment resistance (de la Vega et al., 2018). This also reflects the possible differences in molecular mechanisms in different organizations and different periods.

In conclusion, we identified the positive effect of PZH used locally or by gavage on diabetic wound repair, which involves activating the Nrf2/ARE pathway, improving cell impairment by oxidative stress, enhancing the expression of growth factors in wounds, and possibly reducing the level of blood inflammatory factors in the diabetic mouse wound model to promote wound healing. Our results provide experimental evidence for the treatment of diabetic wounds by PZH and provide a theoretical basis for the treatment of other diseases caused by oxidative stress. In addition, due to the complex composition of PZH, the components that indeed play a role in activating Nrf2 need to be further elucidated in future studies.

## Data Availability

The original contributions presented in the study are included in the article/Supplementary Materials, further inquiries can be directed to the corresponding author.
