# Supplementary material for: Pien-tze-huang promotes wound healing in streptozotocin-induced diabetes models associated with improving oxidative stress via the Nrf2/ARE pathway
Source: Front Pharmacol. 2023 Jan 12;14:1062664. doi: 10.3389/fphar.2023.1062664 (PMC9878590; doi:10.3389/fphar.2023.1062664)

Supplementary 1

The effects of PZH treatment on diabetic rats and mice blood glucose and body weight. (A) The body weight changes after PZH topical treatment in rats. $^{**}p<0.01$, (n=10) (B) The blood glucose level changes after PZH topical treatment in rats. (n=10). The blood glucose levels exceed the upper limit of the blood glucose meter, we use the maximum value of 33.3mmol/L to express. (C) The body weight between high-fat diet fed mice and regular diet fed mice. $^{**}p<0.01$, $^{***}p<0.001$, $^{****}p<0.0001$ (n=6). (D) The body weight changes after PZH gavage treatment in mice. (n=10). (E) The blood glucose level changes after PZH gavage treatment in mice. (n=10). Data are expressed as the mean ± SD.

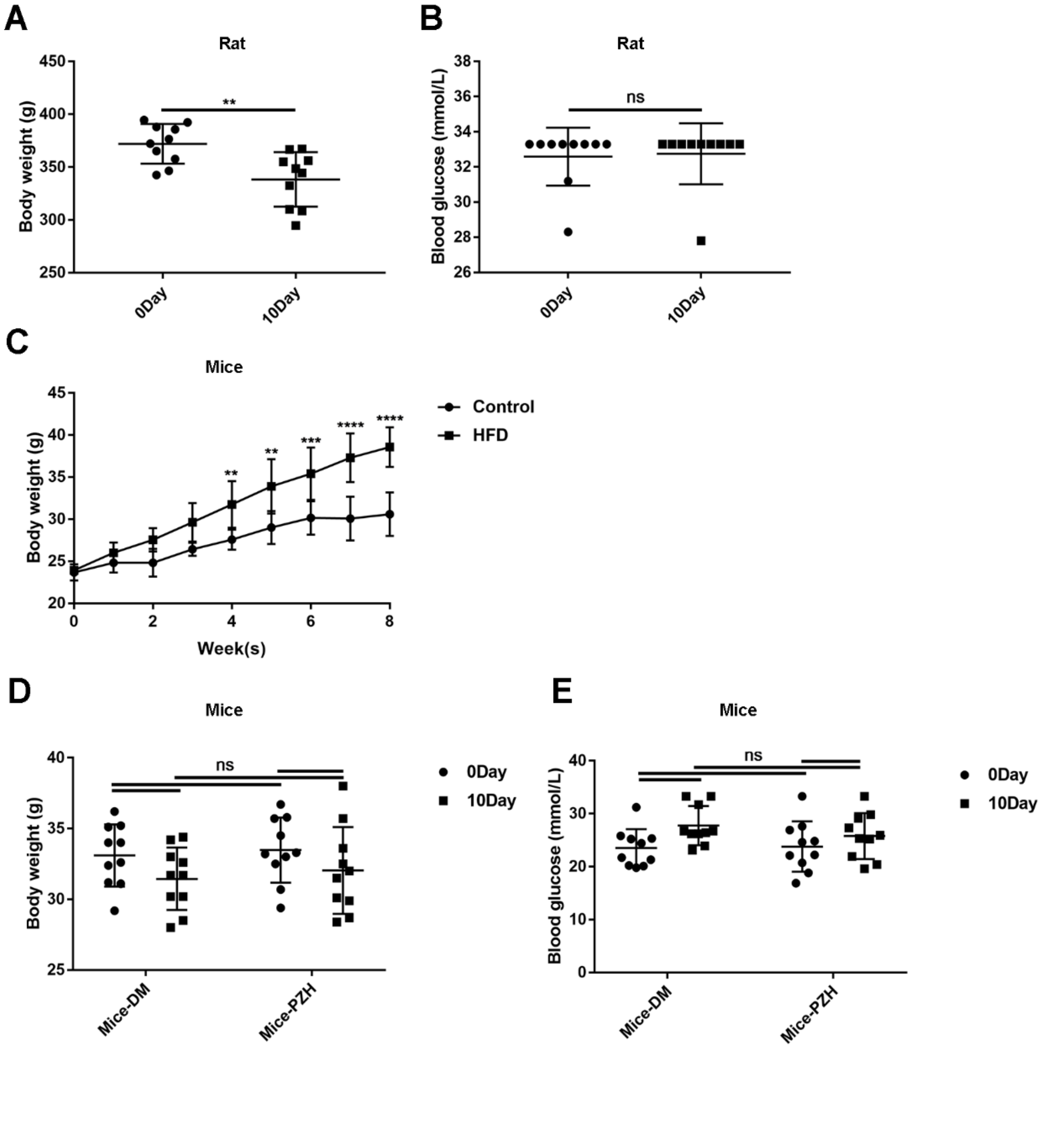

Supplement: Supplementary file 1 [file Image1.pdf]
